# Iterative Bayesian denoising based on variance stabilization using Contourlet Transform with Sharp Frequency Localization: application to EFTEM images

**DOI:** 10.1186/s42490-019-0013-0

**Published:** 2019-06-13

**Authors:** Soumia Sid Ahmed, Zoubeida Messali, Larbi Boubchir, Ahmed Bouridane, Sergio Marco, Cédric Messaoudi

**Affiliations:** 1 Faculty of Science and Technology, Mohamed El Bachir El Ibrahimi University, Bordj Bou Arreridj, Algeria; 2 LIASD research Lab., Department of Computer Science, University of Paris 8, Saint-Denis, France; 3 Department of Computer and Information Sciences, Northumbria University, Newcastle upon Tyne, UK; 4 INSERM, Institut Curie, University of Paris Saclay, Orsay, France

**Keywords:** Image denoising, Variance stabilizing transformation, Bayesian estimation, Contourlet transform, EFTEM

## Abstract

**Background:**

Due to the presence of high noise level in tomographic series of energy filtered transmission electron microscopy (EFTEM) images, alignment and 3D reconstruction steps become so difficult. To improve the alignment process which will in turn allow a more accurate and better three dimensional tomography reconstructions, a preprocessing step should be applied to the EFTEM data series.

**Results:**

Experiments with real EFTEM data series at low SNR, show the feasibility and the accuracy of the proposed denoising approach being competitive with the best existing methods for Poisson image denoising. The effectiveness of the proposed denoising approach is thanks to the use of a nonparametric Bayesian estimation in the Contourlet Transform with Sharp Frequency Localization Domain (CTSD) and variance stabilizing transformation (VST). Furthermore, the optimal inverse Anscome transformation to obtain the final estimate of the denoised images, has allowed an accurate tomography reconstruction.

**Conclusion:**

The proposed approach provides qualitative information on the 3D distribution of individual chemical elements on the considered sample.

## Backround

Transmission Electron Tomography (TET) is one of the most widely used methods for structural analysis in biology and is capable to reveal subcellular structures at the nanometric scale. The combination of TET with chemical mapping (such energy filter transmission electron microscopy: EFTEM) gives qualitative information on the distribution of the chemical elements by the generation of 3D chemical maps in the analyzed samples [1] thus overcoming the limitation of 2D maps. In an EFTEM mode, the transmitted electrons lose different energies according to their interaction with the atoms present in the sample. These energies are characteristic of each type of interaction where electron magnetic fields can be used to separate these electrons. Thus, it is possible to construct a filtered image using only those electrons having lost a precise energy. This approach allows for the computation of elemental maps as images calculated after removing the unspecific signals. The inherent presence of low signal-to-noise ratio (SNR) in biological specimens when an EFTEM is performed, remains a major issue to generate high resolution and good quality EFTEM-3D maps. thus limiting the use of 3D chemical mapping in biology. This paper aims to improve the quality of the acquired images by applying denoising approaches respecting the physical significance of the pixel values of EFTEM maps (which represent the number of electrons having lost a characteristic energy) to produce 3D chemical maps of very high quality of the sample to be analyzed. There is much interest in developing novel methods to remove the noise in its different forms from images in such a way that the original image is discernible and the signal quality is not modified. However, existing image-enhancement methods amplify noise when they amplify weak edges since they cannot distinguish noise from weak edges [2, 3]. Here, we extend our preliminary work, by considering more general optimal inverses for the Anscombe transformation in an iterative process. on the other hand, it has been shown that there are two types of noise in electron microscopy [4, 5]. The first one comes from the sensor such as the CCD camera, while the second comes from the inelastic interactions of the electrons beam with the specimen. The noise from the camera is dominant and is modeled as a Poisson process. Therefore, we have assumed that the EFTEM images are corrupted by additive Poisson noise. Therefore, EFTEM images are denoised using a Bayesian denoiser in the Contourlet Transform with Sharp Frequency Localization (CTSD) [6] domain iteratively in order to improve progressively the effectiveness of the Anscombe transformation (i.e. variance stabilizing transformation VST) [7, 8]. Furthermore, we demonstrate that the assumption of a Poisson noise with a combination of a Bayesian denoiser in CTSD domain and the Anscombe transformation allow for a significant enhancement of the chemical map computation which in turn will enhance the 3D reconstructed volume of EFTEM images with a computational cost at worst twice that of our previous non-iterative Bayesian denoiser [9]. We demonstrate through experiments with real EFTEM images contaminated by additive Poisson noise that the performance of the proposed method substantially surpasses that of previously published methods. The proposed method is qualitatively evaluated in an observer study to assess the improvement of 3-D visualizations of EFTEM series and quantitatively in terms of SNR.

This paper is organized as follows: “Results” section defines the evaluation criteria considered and the computed maps including a comparative analysis of the performance of the proposed denoising method in this study with previously published denoising methods [3, 9–12] on different real data sets. Furthermore, numerical experiments in this section are presented to demonstrate the effectiveness of the proposed method over recent denoising approaches. “Discussion” section discusses the performance and effectiveness of the proposed method. Concluding remarks are given in “Conclusion” section. Finally, “Methods” section describes first the EFTEM images used in this work and which are a specific data collected at different energies 650, 680 and 710 eV from a biological sample, namely Fonsecaea pedrosoi. It also describes the propose iterative denoising method for the purpose to perform chemical maps computation and therefore to enhance the quality of the 3D reconstructed volume of the EFTEM images.

## Results

In order to assess the performance of the proposed method described in “Methods” section, a quantitative evaluation has been carried out against our previously published denoising approach[9] including recent denoising methods. For the sake of comparison, we have only chosen denoising methods using the same Bayesian denoiser with the scale-mixture approximation to the alpha-stable prior, called " *α*-stable mixture" in different domains. The three domains that we have considered are the Wavelet transform [13], the Contourlet transform [14] and the CTSD domains, respectively, as shown in the workflow at the end of this paper (Fig. 5). Knowing that the bloc of Hot spot in the workflow represents a pre-processing of removing the aberrant pixels from the EFTEM images using the ImageJ plugin EFTEM-TomoJ [1, 15]. The EFTEM-TomoJ and TomoJ blocs are the plugins under ImageJ used to compute the elemental map and the 3D tomography reconstruction of our tilt series respectively.

**Fig. 1:**
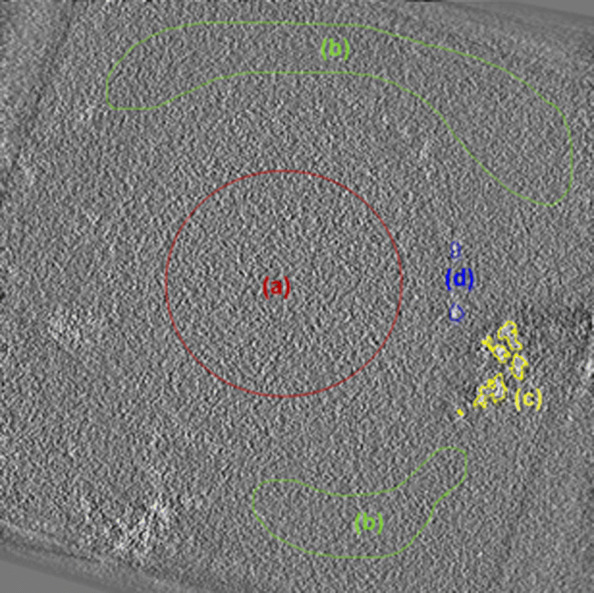
Central plane (number 31 of sections 0 to 63) of the reconstructed volume before denoising. The gray levels are voxels proportional directly to the quantity of the present iron. **a** cytoplasm, **b** resin, **c** iron aggregate area **d** iron aggregates on the cell wall which are considered as the useful signal and is used to evaluate the different algorithms

Since the aim of this study is to enhance the quality of the reconstructed volume of the sample, we have not assessed our proposed method on the 3D volumes for evaluating its effectiveness before and after doing the reconstruction. In addition to the visual quality of the 3D volumes, we have used two evaluations criteria: the SNR and the weber contrast (*C*_*W*_) [16] of the iron aggregates present at the cell wall (signal) in the 3D volume using the resin area as the background. Both the SNR and *C*_*W*_ are calculated using the projections from the central plans (20 to 38) which contains the aggregates in the reconstructed volume. Figure 1 shows the central plan of the reconstructed volume and the different areas before denoising.

The SNR was calculated in decibels using the following equation:

1 $$  SNR_{wall}=10\log_{10}\left[ \left(\frac{\overline{W}-\overline{R}}{\sigma_{resin}}\right)^{2} \right]  $$

where $\overline {W}$ and $\overline {R}$ are the average values of the amplitude of the net wall signal and the resin, respectively, *α*_*resin*_ is the standard deviation of the resin.

**Fig. 2:**
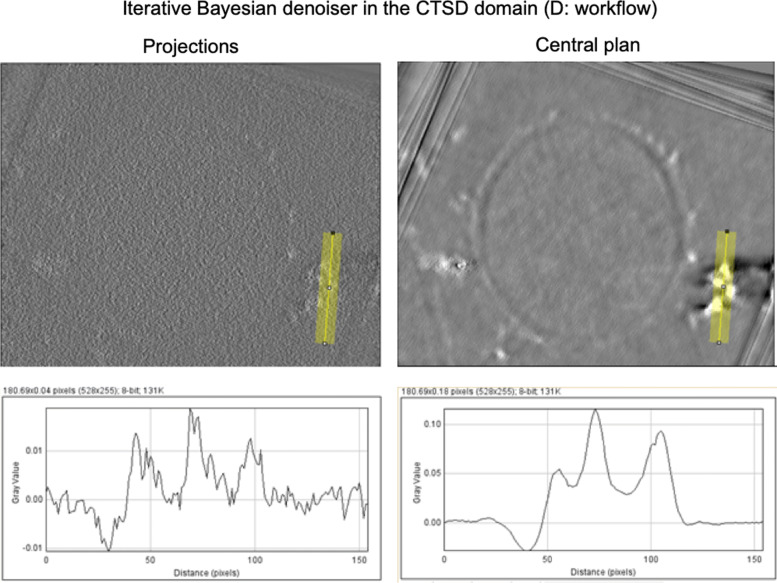
Results of the iterative denoising process on images. The charts correspond to profiles obtained from the lines drawn in each image

**Fig. 3:**
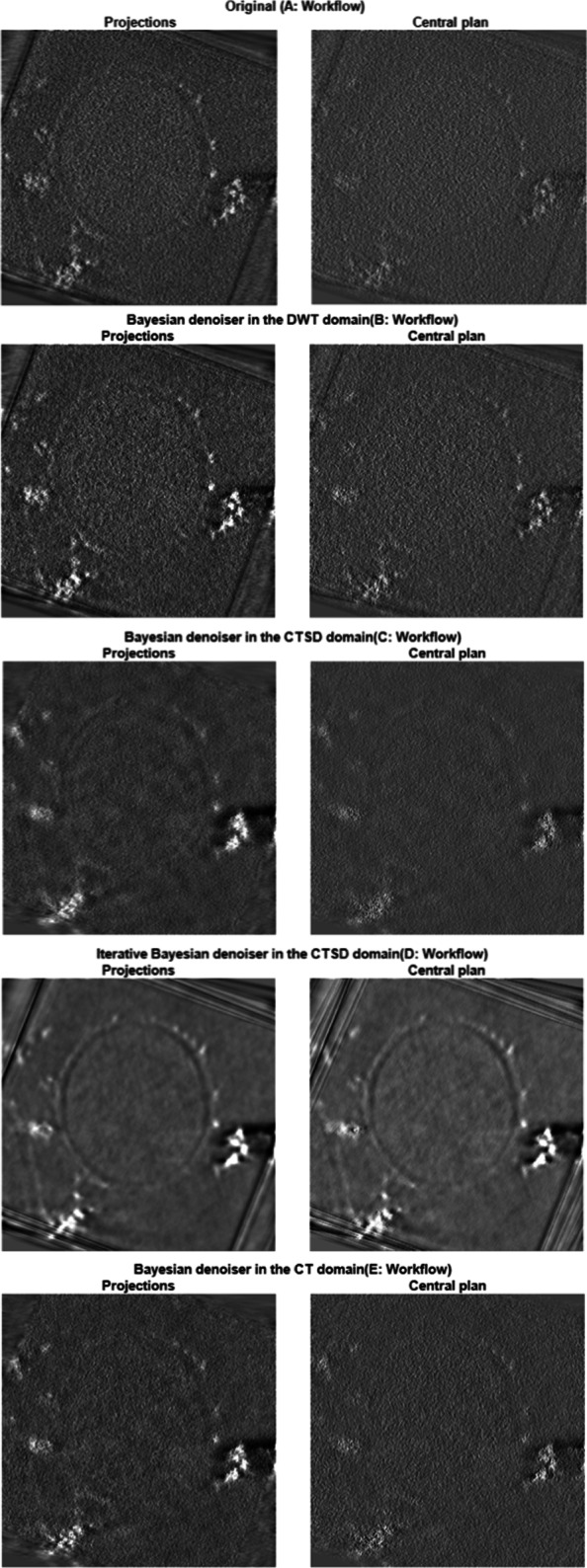
Visual comparisons of the reconstructed volumes, the images are the projection of 18 central plan images and the central image of the 3D volume

In order to calculate the weber contrast in the wall area, we used the following formula:

2 $$  C_{W}=\left[\frac{\overline{W}-\overline{R}}{\overline{R}}\right]  $$

where *C*_*W*_ is the contrast in the wall area, $\overline {W}$ and $\overline {R}$ are the mean values of the pixels in the wall and resin zones, respectively. The detection of the iron aggregate is an important task for further following biologic process. The texture of the different regions in the EFTEM image isn’t considered in this work. Figure 2 shows the visual results of the central plan and the eighteen projections of the reconstructed volume using the estimated images for each denoising method. One can clearly see that the visual quality of the proposed iterative Bayesian denoiser in the CTSD domain with the VST for the Poisson noise outperforms the considered denoising methods. By combining the noisy observation with a previously obtained estimate of the noise free data, our denoiser overcomes the limitations of our original Bayesian denoiser in [9]. The zooming on a textured area of the sample proves not only that our denoiser ensures a good compromise between the noise rejection and the conservation of the finer details in the image, but also there are some details that were hidden due to the noise but after the denoising they became visually clear as shown in Fig. 3.

**Fig. 4:**
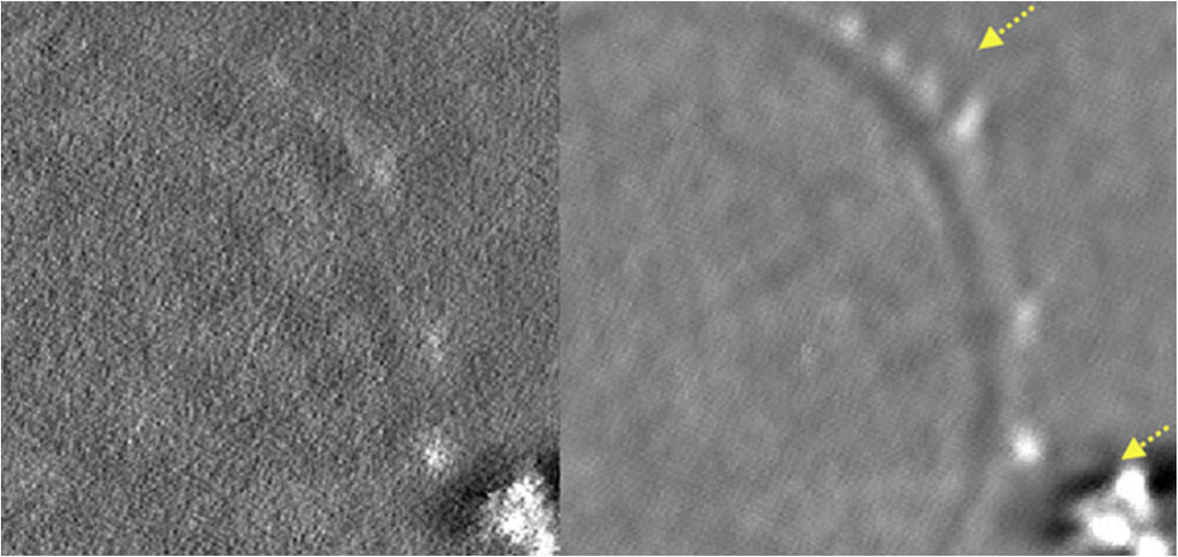
Visual comparison of the projections of the EFTEM images using the iterative Bayesian denoising in the CTSD domain with VST for the Poisson noise and the Bayesian estimator in the CTSD domain [9]. The image was zoomed on a textured area of the *Fonsecaea pedrosoi*, where the yellow arrows indicate the iron aggregates on the cell wall

To demonstrate that the proposed denoising process maintain the contents of the original images, we plot the profile of the images before and after the proposed denoising process, as we did in our previous work [17], using ImageJ 1.48v. In Fig. 4, we plot a 26-pixel integrated intensity profile along the region of interest ROI ’iron aggregate area’ on both original noisy images and denoised images. We clearly observe that the contents of the denoised images are not affected.

**Fig. 5:**
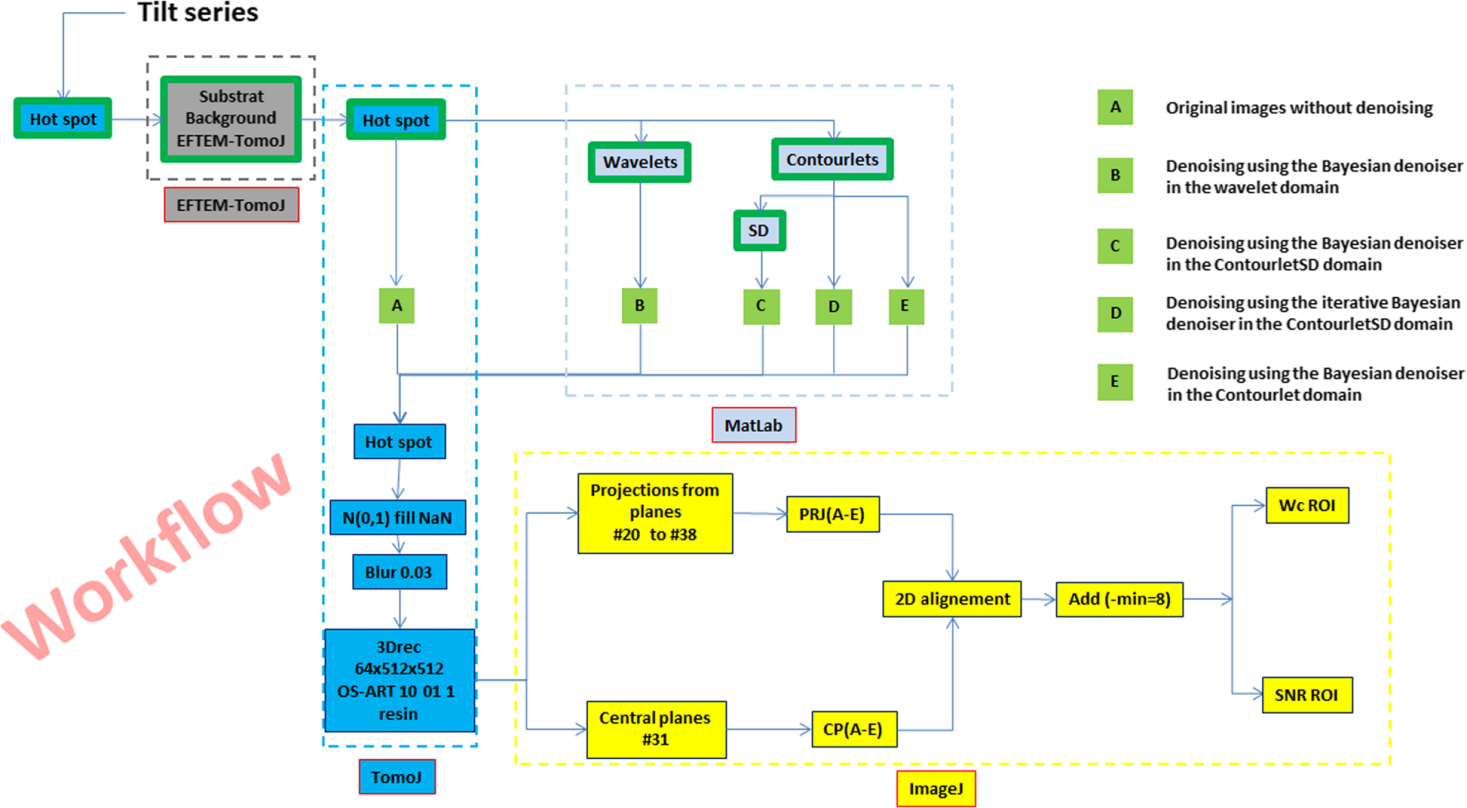
Workflow

Figure 5 summarizes all the methods that we have used in this study where (A) is the reference. We reconstructed the 3D volume of the original images (i.e. without denoising) to compare its quality with the quality of those with denoising. The outputs of (B), (C), (D) and (E) are the tilt series denoised using the Bayesian denoiser in the wavelet, the contourlet SD, the contourlet SD in iterative way and in the contourlet domains, respectively. The bloc of Hot spot in the workflow represents a pre-processing of removing the aberrant pixels from the EFTEM images using the ImageJ plugin EFTEMTomoJ [1, 14]. This step is applied before and after the denoising step to make the alignement process during the reconstruction easier. The EFTEM-TomoJ and TomoJ blocs are the plugins under ImageJ used to compute the elemental map using the 3-window technique which requires three energy-filtered images and the 3D tomography reconstruction of our tilt series, respectively.

To measure performance improvement, we have calculated the SNR (Table 1) and the weber contrast *C*_*W*_ using the reconstructed volumes before and after denoising of the whole database (228 images) for each denoising method, which means 912 images total. After analysing the results, one can see that the SNR and the *C*_*W*_ are enhanced in all the applied methods and the Bayesian estimator in the wavelet and the contourlet transform domains is comparable to the Bayesian estimator in the CTSD domain. One can also notice that the proposed iterative denoiser outperforms the previous methods, especially our previous work [9] and gives much better results in terms of both SNR and *C*_*W*_, where the SNR is enhanced by about 11 dB compared to the Bayesian estimator in the CTSD domain [9]. The main reason is that the iterative combination with a previous estimate refines the stabilization and helps to tackle the problem of the low SNR for this type of images. These findings suggest that the proposed iterative Bayesian denoising in the CTSD domain with VST is an accurate method adapted to capture the fine details that are hidden because of the Poisson noise.

**Table 1:** SNR and contrast *C*_*W*_ of the wall area

Projections	*SNR*_*wall*_	*C*_*W*_	$\overline {W}$	$\overline {R}$	*resin*_*std*_
Original	2.66	0.06	21.30	20.00	0.96
Baysian denoiser in WT domain	9.99	0.10	22.01	19.95	0.65
Baysian denoiser in CTSD domain	8.05	0.07	21.33	19.91	0.56
Baysian denoiser in CT domain	9.01	0.08	21.63	19.91	0.61
**Proposed iterative Bayesian denoiser with VST in CTSD domain**	**19.21**	0.10	22.08	19.95	0.23

(*resin*_*std*_ *denotes the standard deviation of the resin area)*

We should note, that the accurate and judicious assumption of the Poisson distribution instead of the Gaussian one to model the additive noise in the observation data EFTEM, helped to improve the considered Bayesian estimators.

## Discussion

After analysing the results, one can see that the SNR and the CW are enhanced in all the applied methods and the Bayesian estimator in the wavelet and the contourlet transform domains is comparable to the Bayesian estimator in the CTSD domain. One can also notice that the proposed iterative denoiser outperforms the previous methods, especially our previous work [9] and gives much better results in terms of both SNR and CW, where the SNR is enhanced by about 11 dB compared to the Bayesian estimator in the CTSD domain [9]. The main reason is that the iterative combination with a previous estimate refines the stabilization and helps to tackle the problem of the low SNR for this type of images. These findings suggest that the proposed iterative Bayesian denoising in the CTSD domain with VST is an accurate method adapted to capture the fine details that are hidden because of the Poisson noise. We should note, that the accurate and judicious assumption of the Poisson distribution instead of the Gaussian one to model the additive noise in the observation data EFTEM, helped to improve the considered Bayesian estimators.

## Conclusion

This paper has proposed a novel iterative method based on a nonparametric Bayesian estimator in CTSD domain with VST which is capable to denoise EFTEM images. The iterative combination with a previous estimate (denoised image) refines the stabilization which leads to a better quality of the images in terms of a higher SNR and contrast which in turn enhances the 3D tomographic reconstruction. In order to illustrate the potential of the proposed denoising method and analyze the importance of embedding the VST framework within the iterations, we have compared our results using simplified version of the developed algorithm (without iteration and without VST) in different domains with the proposed denoising algorithm. After applying the non iterative Bayesian estimator in the different domains, we have obtained good results where the SNR is considerably enhanced. To further address the problems associated with missing details in the denoised images, we have refined our previous method by taking into account the geometrical information of the images (i.e. contours). Therefore, we have applied iteratively the Bayesian denoiser in the CTSD domain where we have used the Anscombe transform to normalize the image noise. Then denoising the EFTEM images with a nonlinear nonparametric Bayesian estimator is performed to reconstruct the images to their original range via an optimal inverse transformation. This algorithm gave us better results as shown in Fig. 2, where details hidden after previous denoising approach, are now preserved, as shown in Fig. 3. Our future will focus on studying other nonparametric Bayesian estimators, in particular, the estimator based on Bessel-K-form (BKF) density [18–20].

## Methods

### Nature of data

**Fig. 6:**
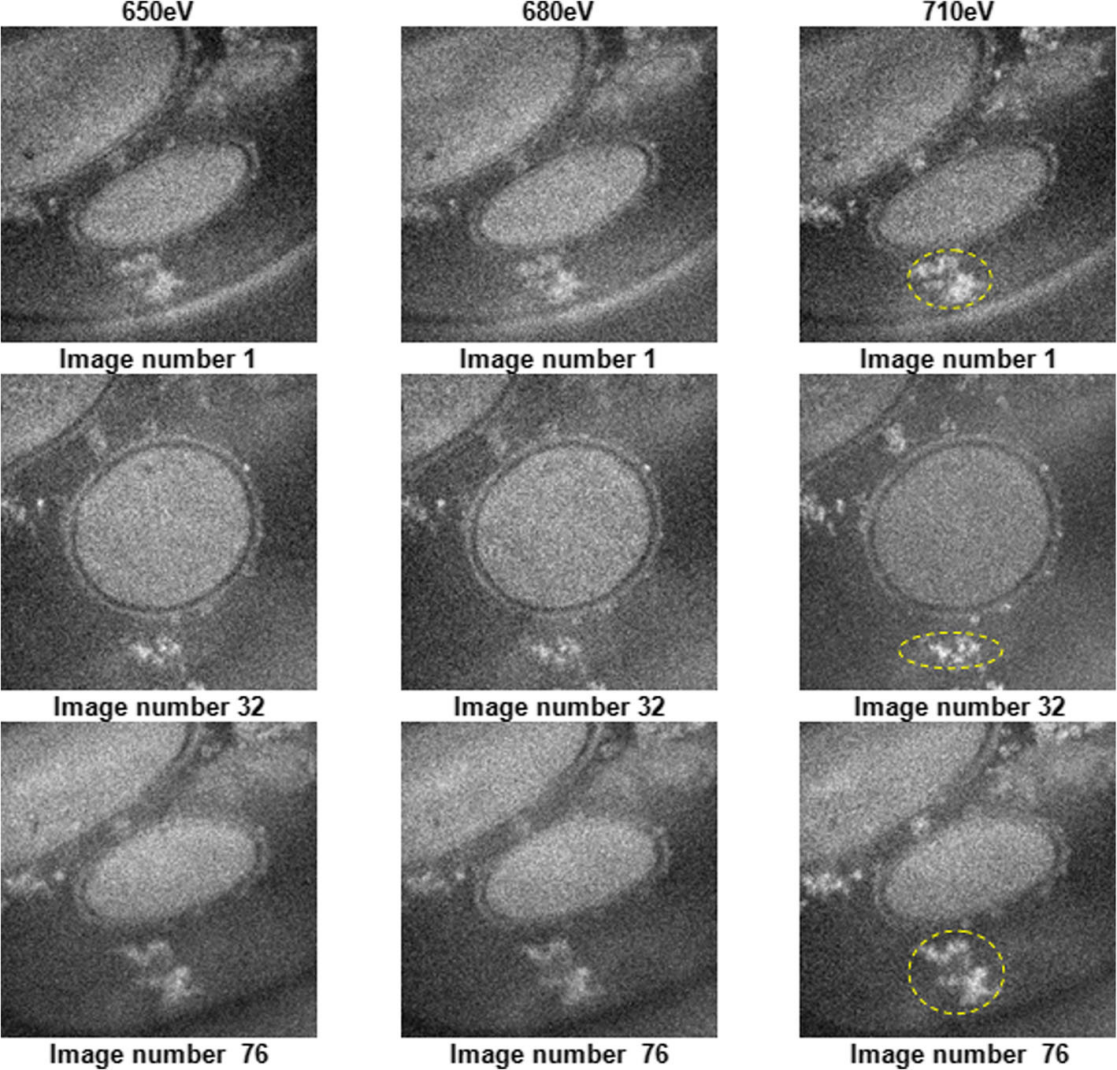
Example images (tilt angles of −60^∘^,0^∘^ and 60^∘^) for tomographic EFTEM series acquired at 650, 680 and 710 eV

The denoising methods were applied on experimental data collected from a biological sample (Fonsecaea pedrosoi). These experimental data consist of EFTEM tomographic tilt series acquired using a Saxton scheme from −60^∘^ to 60^∘^ with TEMography Software from JEOL Ltd (interested readers are referred to [1]. In our case, we have used three series of different energies 650, 680 (corresponding to pre-edges representing the background of the chemical element Fe) and 710 eV (corresponding to the Fe L2 peak representing the characteristic iron signal) with an energy window of 20 eV; each one containing 76 gray-scale images of size 512 ×512 pixels each. Figure 6 shows three examples of images number 1, 32 and 76 from each series at different energies (650, 680 and 710 eV) and three angles (−60^∘^,0^∘^ and 60^∘^). Three principal image areas are considered in quantitative assessments, namely: (a) cytoplasm, (b) resin, (c) iron aggregate area and iron aggregates on the cell wall. The yellow circles in the 710 eV images corresponds to iron aggregates, which are considered as the useful signal and are used to evaluate the different algorithms.

### Proposed denoiser

**Fig. 7:**
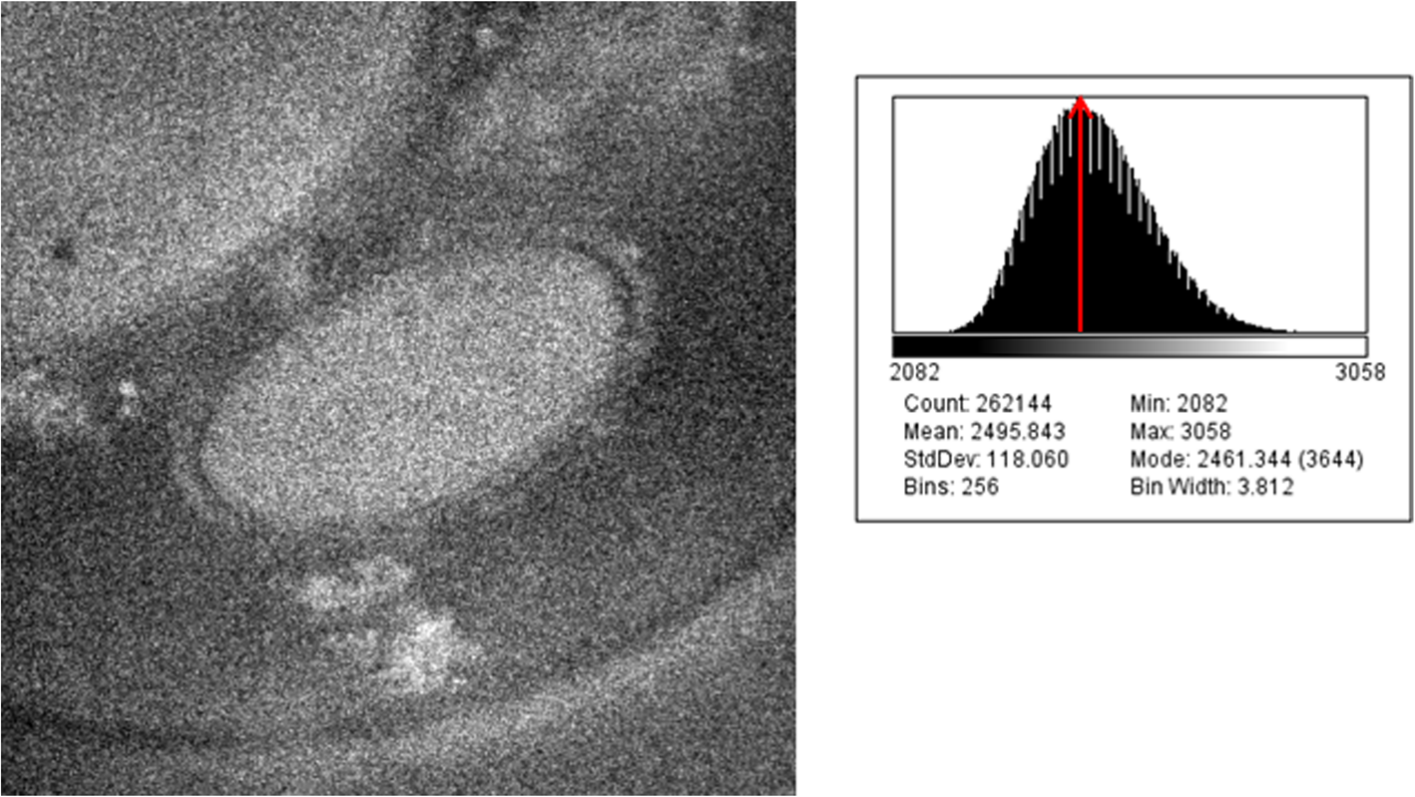
Histogram of EFTEM image *(The EFTEM image number 06 of the 650eV tilt series with its corresponding histogram)*

**Fig. 8:**
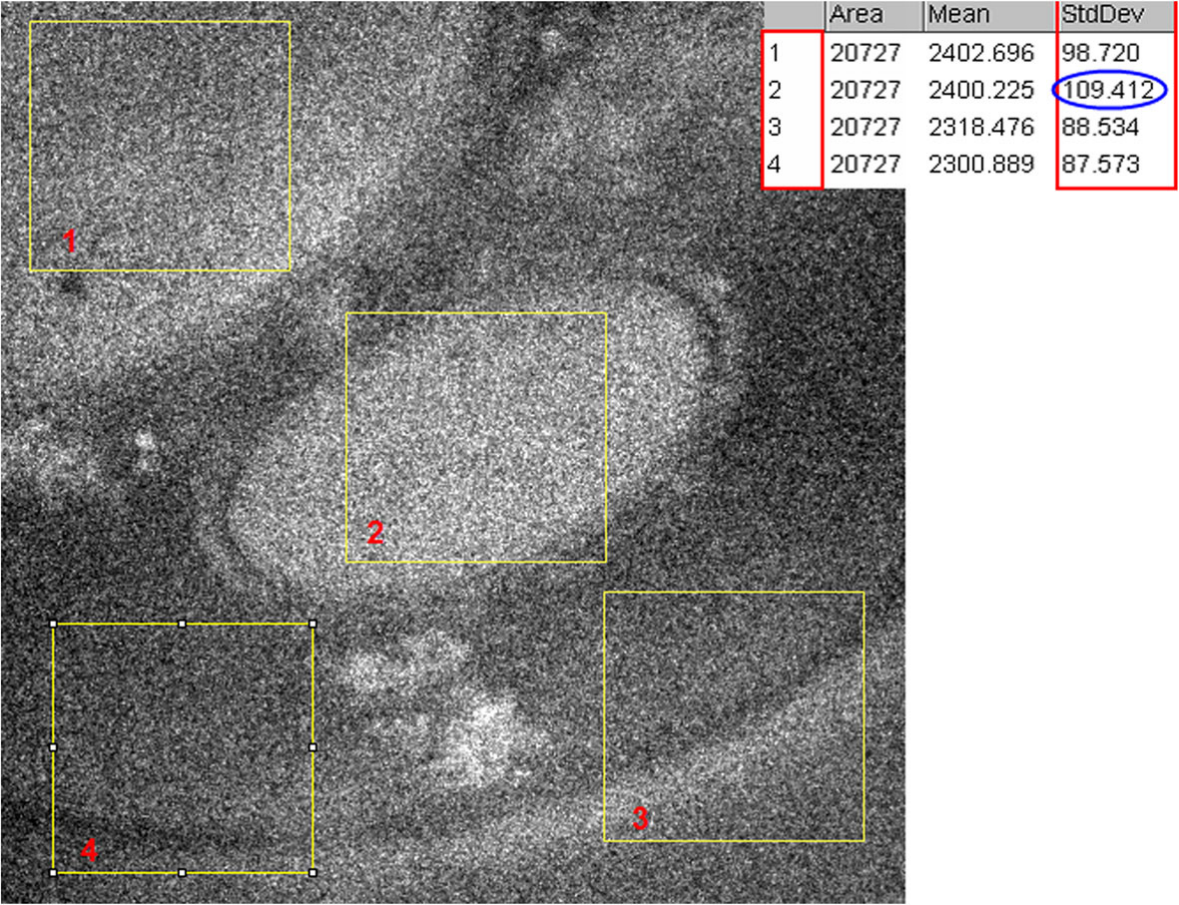
Standard deviation values (STD) in different regions of an EFTEM image

This paper proposes to denoise the EFTEM images using an iterative way. Our inputs are EFTEM images affected by an additive Poisson noise imaged at different energies. The histogram of the noisy images is positively skewed as shown in Fig. 7. To denoise them, we apply a VST approach to standardize the image noise as the first step. Then, we calculate the standard deviation (STD) of different regions in the same image as shown in Fig. 8, in order to confirm that it is not stable as it should be in the case of a Poisson noise. This explains why we need firstly to apply the VST to standardize the image noise. Then, we denoise the images by considering them like they areas being contaminated with an additive white Gaussian noise (AWGN). The iterative proposed algorithm is based on a nonparametric denoising method in the CTSD domain. Once obtaining After getting the denoised images, we apply the optimal inverse of the VST using ; in our case we used the most common one for this purpose which is the Anscombe transformation (AT) [7].

The Anscombe transform converts a Poisson noise to Gaussian noise with variance 1 [7] so, from a mathematical viewpoint, our model is

3 $$  y=x+\varepsilon  $$

where *y* and *x* are respectively the noisy EFTEM image and the original clean image to recover, *ε* is an additive Gaussian noise.

#### Basic assumption

Our input is a noisy EFTEM image *y* composed of pixels *y*(*m,n*), modeled as an independent realization of a Poisson process with parameter *x*(*m,n*)≥0:

4 $$ \begin{aligned} y&(m,n) \sim P(y(m,n)|x(m,n))\\ =&\left\{\begin{array}{ll}\frac{x(m,n)^{y(m,n)}e^{-x(m,n)} }{y(m,n)!} & \quad y \in N \cup\{0\}\\ 0 & \quad elsewhere\\ \end{array}\right. \end{aligned}  $$

knowing that the mean and variance of *y* coincide and are equal to *x*:

5 $$  \mathbb{E}\left\{y|x\right\}=\text{var}\left\{y|x\right\}=x  $$

#### Proposed iterative algorithm

Our goal is to homogenize the noise variance in all image regions. Therefore, we first apply the Anscombe forward transformation to each image. This transformation step normalizes the image noise [21, 22] and yields an image *a*(*y*):

6 $$  a(y)=y_{AT}=2\sqrt{y+3/8}  $$

The observations *a*(*y*) can be treated as corrupted by AWGN with homogeneous variance. After applying AT, we apply a Bayesian denoiser in the CTSD domain (*BD*_*CTSD*_), proposed in our previous work [9] to enhance the observed images in terms of visual quality, contrast and SNR. For the sake of clarity, we first describe the Bayesian denoiser in this section. The transformed observed image is represented in the contourlet-SD domain by:

7 $$ CTSD_{k}(a)=s_{k} + \epsilon_{k}  $$

where *CTSD*_*k*_(*a*),*s*_*k*_ and *ε*_*k*_ are the contourlet coefficients in the *k*^*th*^ directional subband of the observed noisy image, noise-free image and noise respectively.

Because the contourlet has the similar characteristics as the wavelet, so we can straightforwardly extended the Bayesian denoiser proposed in the wavelet domain [11, 12], into the contourlet domain.

In our study, similarly to the wavelet domain, the applied Bayesian denoiser in the contourlet domain is based on adapting a prior statistical model for *s*_*k*_ and then imposes it on the contourlet coefficients to describe their distribution.

In the other hand, it has been shown that the statistical behavior of contourlet coefficients is successfully modeled by families of heavy-tailed distributions such as the *α*-stable. More precisely, Sadreazami et al. [23] demonstrated through the plots of histograms and the computation of kurtosis of the contourlet coefficients that symmetric *α*-stable family, is more appropriate distribution for modeling the contourlet coefficients of natural images than families with exponential tails such as the generalized Gaussian. In view of this, we propose to use the *α*-stable prior with the scale mixture approximation, called " *α*-stable mixture" to model the contourlet subband coefficients [9].

The denoised contourlet coefficients of the image are then estimated by the *L*_2_-based Bayes rules, which correspond to posterior conditional mean (PCM) estimate as shown in our previous work [9]. The inverse contourlet transform is computed through the processed contourlet coefficients to get the denoised image).

The Bayesian denoiser *BD*_*CTSD*_, is viewed as an efficient filter for AWGN. If denoising is ideal, we have:

8 $$  BD_{CTSD}(y_{AT})=BD_{CTSD}(a(y))=\mathbb{E}[y_{AT}|{y}]  $$

The so-called exact unbiased inverse of a [7]

9 $$  I_{a}^{p}: \mathbb{E}\left[a(y)| x\right]\mapsto \mathbb{E}\left[y| x\right]=x  $$

is used to generate the denoised image to the original range of *y*, thus yielding an estimate of *x*:

10 $$  \widehat{x}=I_{a}^{p}(BD_{CTSD}(y_{AT}))  $$

where *BD*_*CTSD*_ denotes the Bayesian denoiser in the CTSD proposed in [9].

The main steps of the proposed denoising algorithm are as follows:

- **Step 1:** Normalize the variance noise of the observed EFTEM data by applying the VST to each image of the three tilt series. This step produces an EFTEM data set such that each image *y*_*AT*_ like it is contaminated with AWGN.
- **Step 2:** Apply the Bayesian denoiser in the CTSD domain (*BD*_*CTSD*_) [9] to the transformed noisy data. The (*BD*_*CTSD*_) consists on: (a) calculate the CTSD coefficients of the *y*_*AT*_, (b) denoise the detail coefficients of the CTSD at each scale and each orientation, (c) reconstruct the denoised image by applying the inverse CTSD to the estimated coefficients. This is done for each image separately. We should recall that for the Bayesian denoiser in the contourlet transform and the contourlet-SD, we selected the number of levels for the Directional Filter Bank (DFB) at each pyramidal level equal to (2, 3, 4, 5) pkva filters and we did not downsample the low-pass subband at the first level of decomposition, based on [6].
- **Step 3:** Apply the optimal inverse AT to generate the denoised image to the original range of *y*.

**Fig. 9:**
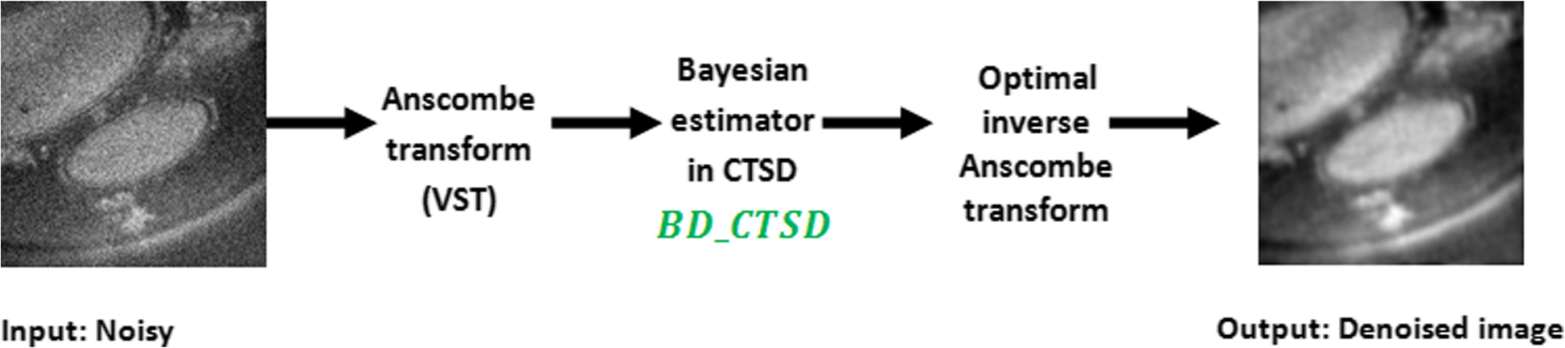
Flowchart of the Bayesian Denoiser in the CTSD domain with Variance Stabilization using Anscombe transform

Figure 9 resumes the steps of the proposed denoising algorithm.

In order to enhance the performance of our proposed denoiser, we follow the same steps as in the paper of Lucio Azzari and Alessandro Foi [7]. We use an iterative algorithm based on convex combination of $\widehat {x}_{i-1}$ and *y*:

11 $$  \overline{y}_{i}=\lambda_{i}y+(1-\lambda_{i})\widehat{x}_{i-1}  $$

where 0<*λ*≤1 and $\widehat {x}_{i}$ is the estimate of $\widehat {x}$ at iteration *i*. *λ* depends on the number of iterations *K* and *λ*_*K*_ and is defined as $\lambda _{i} = 1 - \frac {i-1}{K-1}(1-\lambda _{K})$ where the parameters *K*, *λ*_*K*_ are adaptively selected based on the quantiles of *y* [7]. In the experimental study, all our results have *K*≤4, because there isn’t a significant enhancement of the results in terms of SNR neither of *C*_*W*_ by increasing the number of iterations. Furthermore, the running time of the proposed algorithm increases. We use $\widehat {x}_{i-1}$ instead of the previous $\widehat {x}_{i}$, at each iteration of the algorithm. We apply the Anscombe transformation to image $\overline {y}_{i}$, yielding $f_{i}=a(\widehat {y}_{i})=\widehat {y}_{AT_{i}}$. Then we perform a Bayesian denoising process *BD*_*CTSD*_ to obtain a denoised image $D_{i}= BD_{CTSD}[a(\overline {y}_{i})]$. After getting *D*_*i*_, we return it to its original range by applying the exact unbiased inverse of *f*_*i*_ [24]: We transform the image $\overline {y}_{i}$ to the CTSD domain after applying the Anscombe transform,

12 $$  \widehat{x}_{i}=I_{f_{i}}^{\lambda_{i}}(D_{i})  $$

As in [7], we do the convex combination with a linear binning which can be especially beneficial at the first iterations.

13 $$  \begin{aligned} \widehat{x}_{i}= B_{h_{i}}^{-1} \hspace{85pt} \\ \left[I_{f_{i}}^{\lambda_{i}}\left(BD_{CTSD}\left[f_{i}\left(B_{h_{i}}[{\lambda_{i}}{\times}y+(1-{\lambda_{i}}{\times}\widehat{x}_{i-1}) ]\right) \right]\right.\right] \end{aligned}  $$

**Fig. 10:**
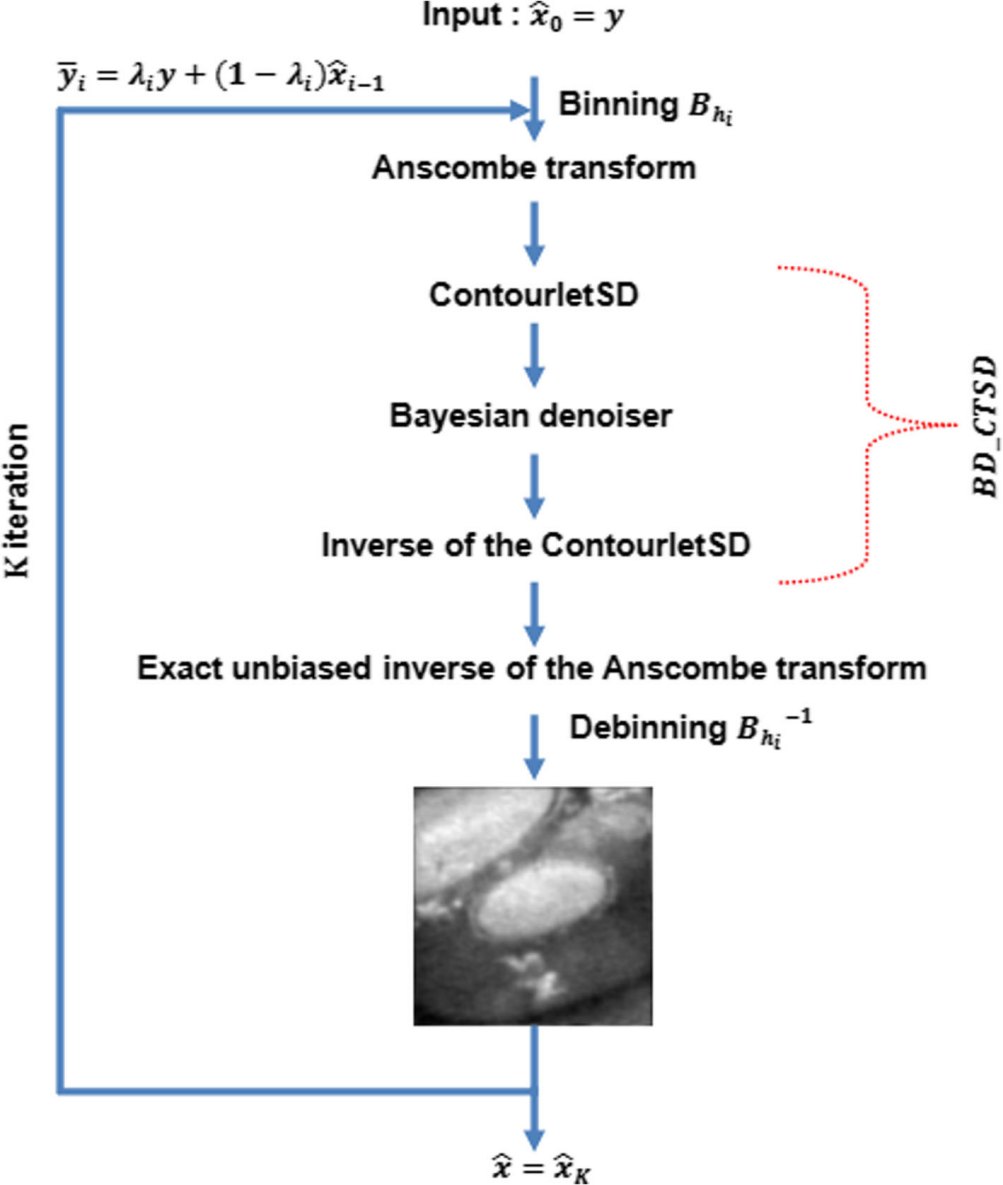
Flowchart of the Bayesian Denoiser with Variance Stabilization using Anscombe Transform

$B_{h_{i}}$ is the binning operator and *h*_*i*_ is the size of the small block at *i*^*th*^ iteration (i.e. bin *h*_*i*_×*h*_*i*_). This operator can be applied to $\overline {y}_{i}$, yielding a smaller image where each bin of *h*_*i*_×*h*_*i*_ pixels from $\overline {y}_{i}$ represents a single pixel equal to their sum. Note that $B_{h_{i}}[\overline {y}_{i}]$ is subject to the same conditional probability of $\overline {y}_{i}$ which means that the adoption of binning does not interfere with the VST [7], neither with *BD*_*CTSD*_ [9]. $B_{h_{i}}^{-1}$ is the inverse binning operator. The entire denoising algorithm is summarized in Fig. 10.

## Abbreviations

AT: Anscombe transformation
AWGN: Additive white Gaussian noise
CTSD: Transform with sharp frequency localization
DFB: Directional filter bank
EFTEM: Energy filter transmission electron microscopy
SNR: Signal-to-noise ratio
STD: Standard deviation
TET: Transmission electron tomography
VST: Variance stabilizing transformation
